# Multigenic Families in Ichnovirus: A Tissue and Host Specificity Study through Expression Analysis of Vankyrins from ***Hyposoter didymator*** **Ichnovirus**

**DOI:** 10.1371/journal.pone.0027522

**Published:** 2011-11-08

**Authors:** Gabriel Clavijo, Tristan Dorémus, Marc Ravallec, Marie-Anne Mannucci, Véronique Jouan, Anne-Nathalie Volkoff, Isabelle Darboux

**Affiliations:** 1 INRA, UMR 1333- Diversité, Génomes et Interactions Microorganismes-Insectes, Montpellier, France; 2 Université Montpellier 2, UMR 1333- Diversité, Génomes et Interactions Microorganismes-Insectes, Montpellier, France; University of Kansas Medical Center, United States of America

## Abstract

The viral *ankyrin* (*vankyrin*) gene family is represented in all polydnavirus (PDVs) genomes and encodes proteins homologous to I-kappaBs, inhibitors of NF-kappaB transcription factors. The structural similarities led to the hypothesis that vankyrins mimic eukaryotic factors to subvert important physiological pathways in the infected host. Here, we identified nine *vankyrin* genes in the genome of the *Hyposoter didymator* Ichnovirus (HdIV). Time-course gene expression experiments indicate that all members are expressed throughout parasitism of *Spodoptera frugiperda*, as assessed using RNA extracted from whole larvae. To study tissue and/or species specificity transcriptions, the expression of HdIV *vankyrin* genes were compared between HdIV-injected larvae of *S. frugiperda* and *S. littoralis*. The transcriptional profiles were similar in the two species, including the largely predominant expression of *Hd27-vank1* in all tissues examined. However, in various insect cell lines, the expression patterns of HdIV *vankyrins* differed according to species. No clear relationship between *vankyrin* expression patterns and abundance of *vankyrin*-bearing genomic segments were found in the lepidopteran cell lines. Moreover, in these cells, the amount of *vankyrin*-bearing genomic segments differed substantially between cytosol and nuclei of infected cells, implying the existence of an unexpected step regulating the copy number of HdIV segments in cell nuclei. Our *in vitro* results reveal a host-specific transcriptional profile of *vankyrins* that may be related to the success of parasitism in different hosts. In *Spodoptera* hosts, the predominant expression of *Hd27-vank1* suggests that this protein might have pleiotropic functions during parasitism of these insect species.

## Introduction

Polydnaviruses (PDVs) are obligate symbionts of various wasp endoparasitoids. They play a central role in the success of parasitism by protecting the egg and wasp larva from the host immune system and by influencing host development. PDVs are classified into two genera, Ichnoviruses (IVs) and Bracoviruses (BVs), according to their association with Ichneumonid or Braconid wasps, respectively. These two genera are the result of independent association events between a virus ancestor and a wasp ancestor [1], [2], [3]. However, they display similarities in life cycles and genome structures suggesting evolutionary convergence of the two genera [4]. In the proviral form, the polydnavirus genome is integrated into the parasitic wasp's genome and transmitted vertically. Viral DNA replication and virion formation take place in the calyx cells of the ovary of the wasp. The packaged PDV genome consists of several segments of double-stranded circular DNA of various molecular weights and molar ratios. The PDV particles are transferred to lepidopteran larvae during oviposition. There is no replication of viral DNA in the parasitized host, but the PDVs rapidly infect many cell types and host tissues, including hemocytes and fat body, providing active immunosuppression in the parasitized host, a condition required for the survival of the parasitoids [5], [6], [7], [8], [9].

The genomes enclosed in the PDV particles contain members of several multigene families which differ between BV and IV [4], [10]. The total number of variants present in each gene family differs between PDVs. Although the functional significance of these multiple variants in a gene family has not been fully elucidated, it has been suggested that this phenomenon may reflect functionally diverse temporal and/or tissue-specific expression patterns [11], [12], [13], [14]. The variants may also contribute to allowing a broad host spectrum [15].

Members of the *I-kappaB-like* or *viral ankyrin* (*vankyrin*) multigene family have been found in all sequenced IV an BV genomes [16], [17], [18], [19]. The presence of this family in evolutionarily unrelated viruses strongly suggests that the corresponding proteins target important biological function of the parasitized host. *Vankyrin* family genes encode proteins similar to I-kappaBs, inhibitory proteins which regulate the NF-kappaB signal transduction cascade in insect and mammalian innate immunity [20]. The vankyrin proteins contain ankyrin-repeat domains (ARD) essential for mammalian IkappaB/NF-kappaB binding but lack domains involved in the regulation of the I-kappaB activity. These structural differences have led to the attractive hypothesis that these viral proteins interfere with the normal physiology of the host through molecular mimicry and thereby form irreversible complexes with host NF-kappaBs preventing expression of NF-kappaB-responsive genes [19], [21], [22]. Indeed, some vankyrin proteins from BV are probably involved in immune suppression [19], [21]. IV vankyrin proteins share structural similarities with BV vankyrins, so at least some of them may similarly suppress NF-kappaB activity in parasitized lepidopteran hosts. Previous studies have shown differences in the expression of *vankyrin* genes from *Campoletis sonorensis* IV (CsIV) in parasitized host tissues, allowing division of the genes into two subclasses: those that target host fat body and those that target host hemocytes. In each tissue, vankyrin proteins are targeted to either the cytoplasm or the nucleus. These various findings suggest a functional divergence among the IV vankyrins [12], [22].

Here, we report the sequences of nine members of the *vankyrin* gene family from *Hyposoter didimator IV* (HdIV), the PDV associated with the ichneumonid *H. didymator*. This parasitoid is a generalist wasp that parasitizes and develops in several noctuid species, particularly in *Spodoptera* species. One explanation for the existence of various members in the *vankyrin* gene family of HdIV is that they have different specific functional activities depending on the insect species and/or tissue. To investigate this possibility, we studied the localization of HdIV *vankyrin* transcripts in various tissues and insect species.

## Results

### Identification of the *vankyrin* gene family in HdIV

**Figure 1:**
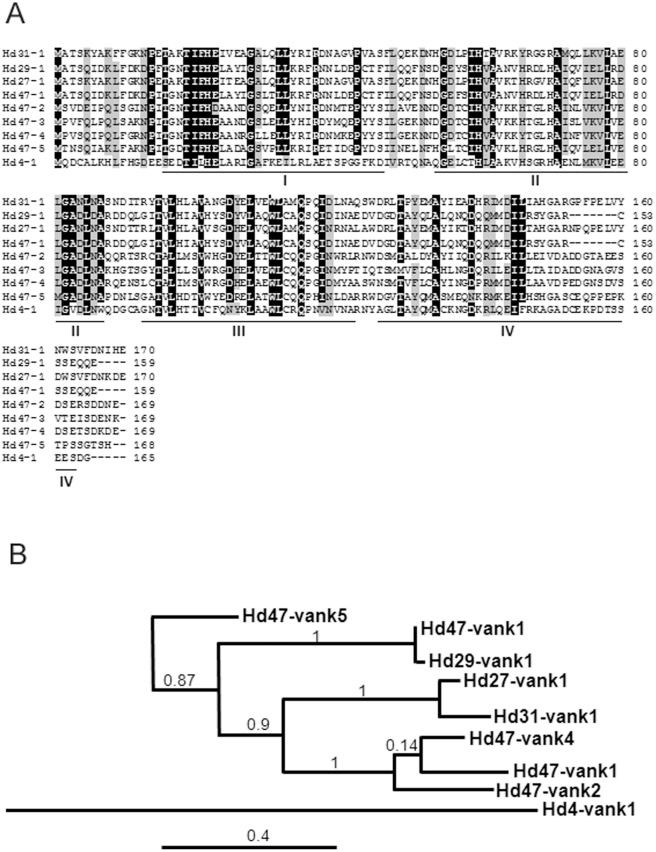
Protein and phylogenetic analysis of HdIV vankyrins. (A) Multiple sequence alignment of the deduced HdIV vankyrin protein sequences obtained with CLUSTALW. Identical and similar amino acids are indicated by dark and gray shading, respectively. Conserved ANK domains are underlined and individual ANK motifs are indicated by roman numerals. The full-length protein sequences have been deposited in GenBank under accession numbers: JF825856 (Hd4-vank1), JF825857 (Hd31-vank1), JF825858 (Hd29-vank1), JF825859 (Hd27-vank1), JF825860 (Hd47-vank1), JF825861 (Hd47-vank2), JF825862 (Hd47-vank3), JF825863 (Hd47-vank4), JF825864 (Hd47-vank5).(B) Phylogenetic analysis of the HdIV vankyrin gene family. The scale bar represents 0.4 substitutions per nucleotide. Numbers at the nodes indicate bootstrap values (%).

The genome of HdIV has been partially sequenced (Volkoff *et al*., unpub.). We identified nine distinct genes sharing overall sequence homology with eukaryotic I-kappaB-like genes and PDV *vankyrins*. The nine genes are distributed on five genome segments named Hd4, Hd27, Hd29, Hd31 and Hd47. Five genes are clustered on the segment Hd47 and were named *Hd47-vank1* to *Hd47-vank5*. The other genes are each on different segments, and were named *Hd4-vank1*, *Hd27-vank1, Hd29-vank1* and *Hd31-vank1*. Their gene structures are similar to each other including the absence of introns and similar lengths of the open reading frames (between 477 to 525 nucleotides). They encode proteins of 159 to 175 amino acids, which share the structural characteristics observed in other PDV vankyrin proteins, and in particular have an ankyrin domain composed of three to four ankyrin repeats and no signal peptide. The percentage of identity between the nine *vankyrin* ranges from 29.4 to 98.7% at the amino acid level (Table 1). The highest percentage of identity is between Hd29-vank1 and Hd47-vank1: the nucleotide sequences differ at only two positions which alter the encoded amino acids. A phylogenetic tree was constructed from the alignment of the deduced protein sequences and revealed three major clusters, with Hd4-vank1 and Hd47-vank5 being located on independent branches (Figure 1). According to the criteria of Friedman and Hughes [23], we identified three *vankyrin* gene duplications. One of the gene duplicates, of which there were three copies on segment Hd47, is entirely consistent with tandem gene duplication. The two other gene duplications included genes located on different segments, less consistent with tandem duplication.

**Table 1:** Identity/similarity (%) matrix for the nine predicted HdIV vankyrin amino acid sequences. Molecular weights (kDa) are indicated.

	SIMILARITY								
	Hd4-vank1	Hd31-vank1	Hd27-vank1	Hd29-vank1	Hd47-vank1	Hd47-vank2	Hd47-vank3	Hd47-vank4	Hd47-vank5
**Hd4-vank1**		47.7	48.2	52.0	55.2	50.0	50.9	51.2	59.5
**Hd31-vank1**	29.4		89.4	57.1	57.1	55.8	55.2	59.2	61.3
**Hd27-vank1**	31.2	82.9		58.7	58.7	56.1	56.7	62.8	61.3
**Hd29-vank1**	34.3	39.4	42.4		99.4	50.9	35.7	53.5	61.9
**Hd47-vank1**	36.4	39.4	42.4	98.7		51.5	53.2	54.1	65.5
**Hd47-vank2**	30.2	42.4	43.3	35.5	36.1		76.9	78.1	60.5
**Hd47-vank3**	32.5	41.3	45.1	52.6	36.3	66.9		83.7	57.1
**Hd47-vank4**	30.8	47.1	49.4	40.1	40.7	68.1	71.0		60.2
**Hd47-vank5**	40.5	41.6	44.5	48.8	49.4	43.6	44.6	46.2	
	**Hd4-vank1**	**Hd31-vank1**	**Hd27-vank1**	**Hd29-vank1**	**Hd47-vank1**	**Hd47-vank2**	**Hd47-vank3**	**Hd47-vank4**	**Hd47-vank5**
	**IDENTITY**								
	**Hd4-vank1**	**Hd31-vank1**	**Hd27-vank1**	**Hd29-vank1**	**Hd47-vank1**	**Hd47-vank2**	**Hd47-vank3**	**Hd47-vank4**	**Hd47-vank5**
**MW (kDa)**	**19.6**	**19.3**	**19.1**	**18.0**	**18.0**	**19.1**	**18.7**	**18.9**	**18.7**

### Spatio-temporal expression of the HdIV *vankyrin* transcripts in parasitized *S. frugiperda* :

**Figure 2:**
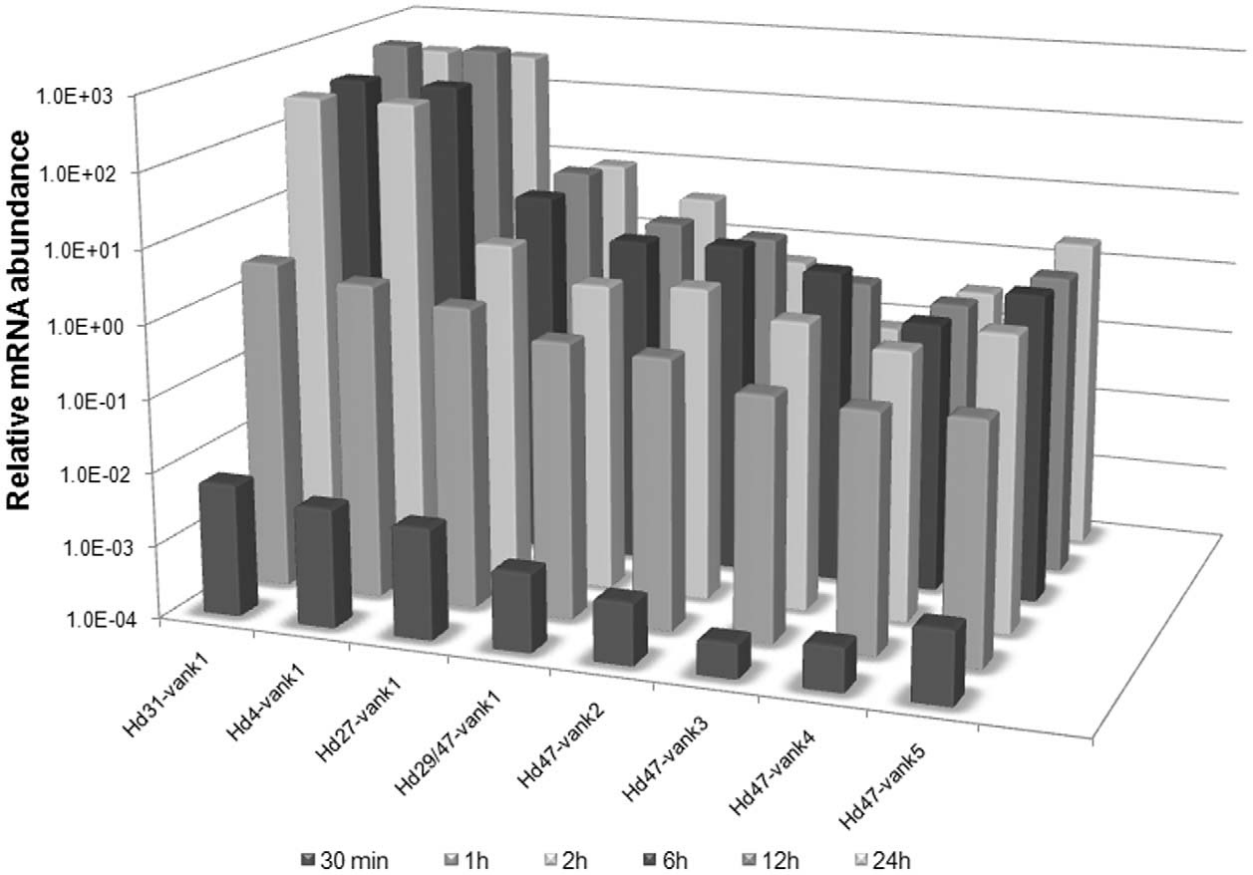
Expression profiles of vankyrins in third-instar parasitized larvae of S. frugiperda. Relative mRNA abundances were measured by real-time RT-PCR and normalized to the values for the housekeeping genes ATP synthase and Ubiquitin E2. The transcripts were extracted from whole larvae over the first 24 h of parasitism. All values are the means for three separate experiments.

**Figure 3:**
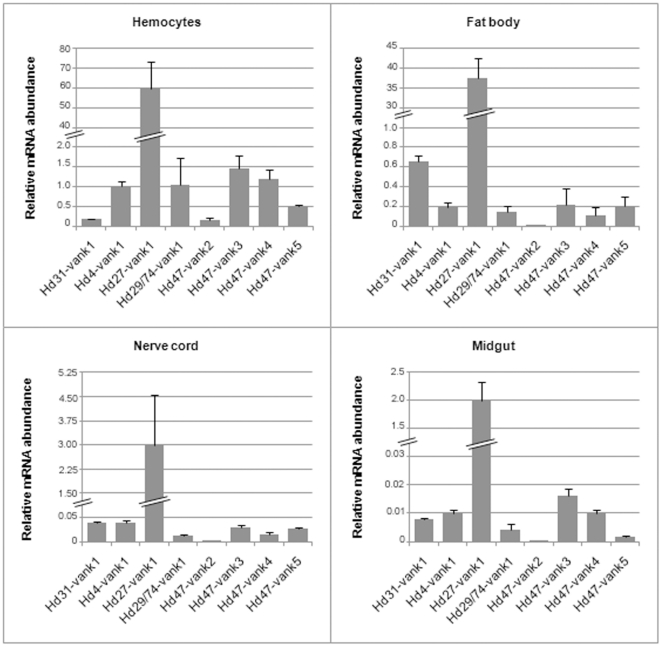
Expression profiles of *vankyrins* in various tissues of parasitized *S. frugiperda* larvae. Expression levels of the HdIV *vankyrin* genes in hemocytes, fat body, nerve cords and midguts of parasitized larvae 24 h p.p. were determined by qRT-PCR. No viral-specific amplification was detected for cDNA pools from non-parasitized tissues (not shown).

The expression of the HdIV *vankyrin* genes was quantified in *S. frugiperda* larvae at various times post-parasitism (30 min to 24 h post-parasitism (p.p.)) by a qRT-PCR analysis using primers specific for each gene (Table S1). Figure 2 illustrates the relative mRNA levels of the HdIV *vankyrin* genes after normalization during the first day p.p. All transcripts were detected at the first time point (30 min p.p.). The abundance of the mRNAs increased slightly during the first two hours, and was then maintained at relatively constant level until the end of the experiment (24 h p.p.). Expression analysis over a longer period (6 days p.p.) indicated that HdIV *vankyrin* transcripts were also present at late stages of parasitism (data not shown). *Hd31-vank1*, *Hd4-vank1* and *Hd27-vank1* mRNAs were significantly more abundant than the other HdIV *vankyrin* mRNAs in the parasitized host. To describe the tissue-specific expression profiles of *vankyrin* members, we performed qRT-PCR using total RNA from several different tissues from third-instar *S. frugiperda* larvae at 24 h p.p. (Figure 3). Almost all *vankyrin* genes were transcribed in all tissues examined, although the extent of transcript accumulation differed. *Hd27-vank1* was the most strongly expressed in all tissues analyzed. The expression profiles of the other HdIV *vankyrins* differed between tissues.

### *Vankyrin* gene expression profiles in two *Spodoptera* species

**Figure 4:**
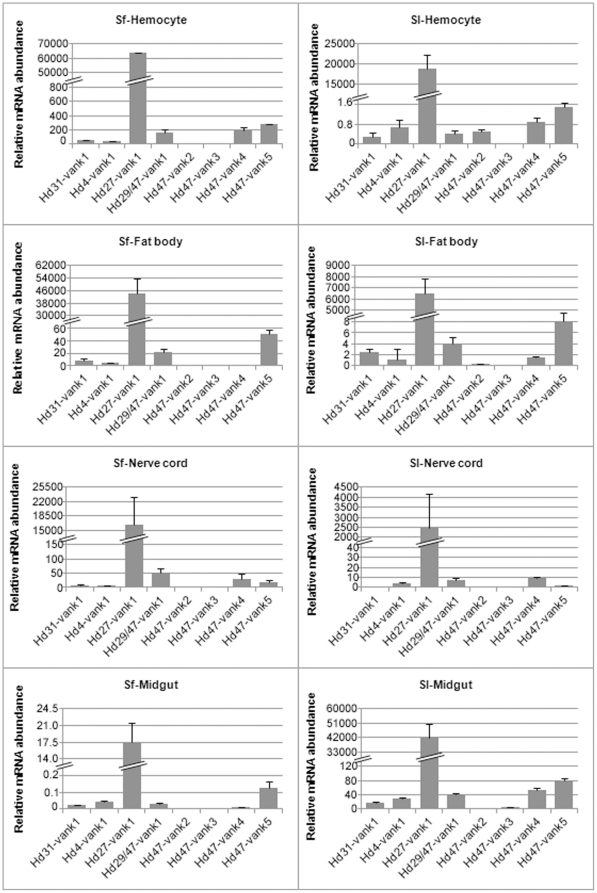
Comparison of expression profiles of *vankyrins* between *S. frugiperda* and *S. littoralis* larvae. Expression levels of the HdIV *vankyrin* genes were determined 24 h p.i. by qRT-PCR in various infected tissues (hemocytes, fat body, nerve cords and midguts) of *S. frugiperda* and *S. littoralis*. *Hd27-vank1* was the most strongly expressed in both species. All values are the means +/− standard deviations from three separate experiments. No viral-specific amplification was detected for cDNA pools from non-infected tissues (not shown).

**Figure 5:**
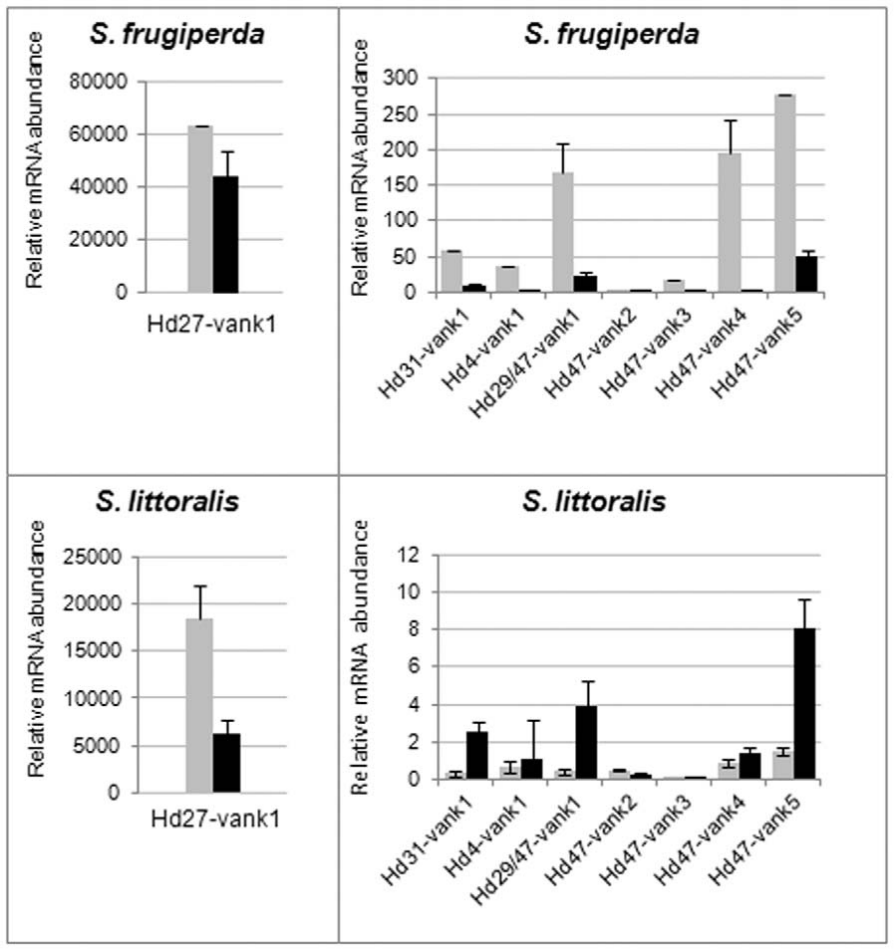
Comparison of expression levels of *vankyrins* between hemocytes and fat body from HdIV-injected *Spodoptera* larvae. Preferential expression of *vankyrins* was detected in hemocytes from *S. frugiperda* and in fat body from *S. littoralis*. Values for *vankyrins* are normalized to those for housekeeping genes (ATP synthase and Ubiquitin E2 in *S. frugiperda* tissues and GADPH and ELF1 in *S. littoralis* tissues). Values for hemocytes are given by gray bars, and fat body as black bars.

To look for potential relationships between host range specificity and HdIV *vankyrin* gene expression patterns, we compared the transcriptional profiles of HdIV *vankyrins* in two closely related *Spodoptera* species. Fifth instar *S. frugiperda* and *S. littoralis* larvae were injected with HdIV. Total RNA was isolated from various tissues 24 h post-injection (p.i.) and subjected to qRT-PCR (Figure 4). In both species, *Hd27-vank1* was more strongly expressed than all other *vankyrins* in all tissues. This finding is consistent with the predominant expression of this gene in the tissues of parasitized *S. frugiperda* larvae. *Hd29/47-vank1*, *Hd47-vank4* and *Hd47-vank5* transcripts were also abundant, although less so than the *Hd27-vank1* transcript in both species. *Hd47-vank2* and *Hd47-vank3* were the most weakly expressed. The expression profiles of HdIV *vankyrin* genes were generally similar in the two *Spodoptera* species, with a few exceptions. For example, *Hd47-vank2* transcripts were detected only in hemocytes of *S. littoralis* and *Hd47-vank4* transcripts accumulated only in midgut cells of *S. littoralis* (Figure 4). The main difference between *S. frugiperda* and *S. littoralis* concerned differences in the expression profiles of HdIV *vankyrins* between hemocytes and fat body (Figure 5). All *vankyrin* genes were preferentially expressed in the hemocytes of *S. frugiperda*. By contrast, fat body was the major site of accumulation of almost all *vankyrin* transcripts in *S. littoralis*.

### Expression profiles of HdIV *vankyrins* in different insect cell lines

**Figure 6:**
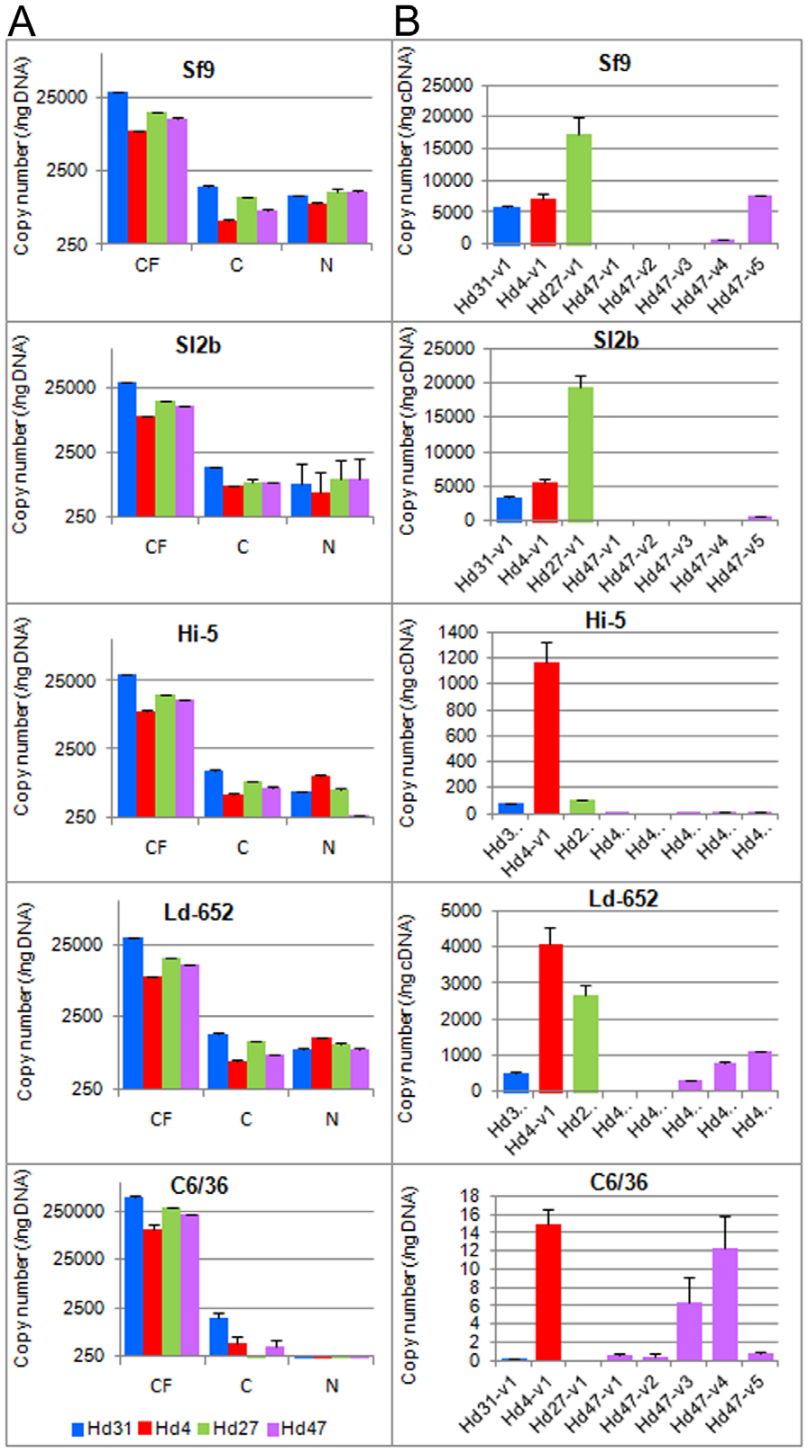
Species-specificity of *vankyrin* expression in various insect cell lines. (A) Absolute qPCR analysis was performed to assay the four HdIV segments in calyx fluid (CF), cell cytoplasm (C) and nuclear (N) extracts from HdIV-infected insect cell lines. The copy number of each DNA segment (i.e. Hd31, Hd4, Hd27 and Hd4) was calculated using a standard curve with primers specific for *Hd31-vank1*, *Hd4-vank1*, *Hd27-vank1* and *Hd4-vank1*, respectively. (B) Absolute qRT-PCR experiments were performed to analyze the expression levels of *vankyrin* genes in selected cell lines at 24 h p.i. Results were normalized to ng of DNA. No viral-specific amplification was detected for cDNA pools from non-infected cell lines (not shown). The detection level of the *vankyrin* mRNAs in C6/36 cells was close to those found in the non-infected conditions, indicating that there is no *vankyrin* gene expression in the dipteran cell line.

To extend our description of host-specific patterns of HdIV *vankyrins* expression, we studied for the transcriptions in various different insect cell lines infected with HdIV. Four different lepidopteran and one dipteran cell lines were used (see Methods for details). Prior to this analysis, we tested the capacity of HdIV particles to infect the selected cell lines. We used absolute qPCR to determine the copy number of the four HdIV segments bearing the *vankyrin* genes (i.e. Hd4, Hd27, Hd31 and Hd47) in the calyx fluid, in the cytoplasm and in the nucleus of infected cells at 2 h post-infection (Figure 6A). Differences in the DNA copy number in all four HdIV genomic segments were found between the calyx fluid used to infect the lepidopteran cells and the calyx fluid used to infect the dipteran cells. These differences are probably due to variability in the physiological state of the wasps used for each experiment. However the proportion of the four DNA segments did not vary in all HdIV extractions. The HdIV segments were present in non-equimolar numbers in calyx fluid (Kruskal-Wallis chi-squared  =  8.54, df = 3, p-value = 0.03608), with segment Hd31 being more abundant than the three others (Figure 6A). In the lepidopteran cells, the copy numbers of the four segments present in the cells (cytoplasm and nucleus) were much lower than that in calyx fluid (8.3 to 12.4 %). In the dipteran cells, the efficiency of HdIV entry into the cells was considerably lower (0.2 %), indicating that the mosquito cells are less permissive to HdIV infection than lepidopteran cells. In all insect cell lines, the respective proportions of each segment in cytosol were roughly the same as in calyx fluid (Figure 6A). The four segments were also detected in the cell nucleus, where the viral transcription occurs (Figure 6A). However, we observed obvious differences in genomic DNA abundance between cytosol and nuclei in the lepidopteran cell lines, suggesting species-specificity selectivity in viral entry. In the spodopteran cells and in Ld-652 cells, relative abundance of the Hd31 segment in the nuclei was lower, such that the copy numbers of the four segments were relatively similar. In the *Trichoplusia ni* (High Five) cells, the relative copy number of Hd4 was higher in nuclei (41 %) than cytosol (17 %), whereas that of Hd31 was higher in the cytosol (37 %) than nuclei (23 %).

The expression profiles of the HdIV *vankyrin* genes were analyzed at 24 h p.i. in all insect cell lines (Figure 6B). In Sf9 and Sl2b, *Hd27-vank1* transcripts were abundant and *Hd47-vank1-4* were only weakly expressed. This expression pattern is consistent with those observed in *Spodoptera* larvae. In the High Five cells, more than 85 % of all HdIV *vankyrin* transcripts were *Hd4-vank1* mRNAs, although the Hd4 segment represented 4 1% of the four segments. Conversely, *Hd27-vank1* mRNA made up 7 % of the transcripts, although the corresponding genomic segment was relatively abundant (26 %). In *Lymantria dispar* cells (Ld-652), all HdIV *vankyrin* genes were clearly detected, but the *Hd4-vank1* transcripts accounted for more than 42 % of the total for the *vankyrin* mRNAs. Conversely, *Hd31-vank1* was very weakly expressed (5.6 %), although segment Hd31 was highly abundant in the nuclei (29.2 %). Lastly, expression of HdIV *vankyrin* transcripts was detected in C6/36 dipteran cells at a level similar to those found in control cells, even though we demonstrated that HdIV is able to enter the cell nuclei. Therefore, our results indicate that the relative amounts of the mRNAs of the HdIV *vankyrin* family differed considerably from cell line to cell line, and do so independently of the number of gene copies that reached the cell nuclei.

## Discussion

The availability of complete or almost complete genome sequences has revealed that the *vankyrin* gene family is found in all PDV genomes and represents one of the largest families. We identified nine *vankyrin* genes in the genome of HdIV, similar to the number of genes (11 variants) present in the fully sequenced genome of the closely related species *H. fugitivus* ichnovirus (HfIV) [10]. The HdIV genome is not completely sequenced, so we cannot exclude the possibility that additional variants are present.

Time-course gene expression experiments in parasitized *S. frugiperda* larvae revealed that all HdIV *vankyrin* genes are transcriptionally active, indicating that none of the genes identified are pseudogenes. Expression of each HdIV *vankyrin* member was detected early after parasitization and was maintained thereafter. The amounts of the mRNAs for the various *vankyrin* members differed in the tissues examined, and these differences showed no apparent correlation with the phylogenetic relationships within the gene family. The most obvious example is provided by two closely related genes, *Hd27-vank1* and *Hd31-vank1*, which are expressed at very different levels. The transcriptional patterns of HdIV *vankyrins* as assessed in whole larvae were different to those in samples from various tissues. In whole larvae, *Hd31-vank1* and *Hd4-vank1* were the most abundant HdIV *vankyrin* mRNAs, whereas in the tissues we studied, they were weakly detected, suggesting that the tissues in which *Hd31-vank1* and *Hd4-vank1* are mainly expressed were among those not tested in this study. One of these tissues may be the cuticular epithelium which has been demonstrated to be targeted by ichnoviruses. F1-1, a *Tranosema rostrale* ichnovirus repeat element gene is most abundantly expressed in the cuticular epithelium than the three others tissues examined (midgut, fat body and hemocytes) in *Choristoneura fumiferana* [14].

Our study reveals similar expression profiles for the HdIV *vankyrin* gene family in two species of the same genus, *S. frugiperda* and *S. littoralis*. In particular, in all tissues analyzed from both species, *Hd27-vank1* mRNA was considerably more abundant than other HdIV *vankyrin* mRNAs. The predominant expression of *Hd27-vank1* was also observed in parasitized *S. frugiperda* larvae, Sl2b and Sf9 cells. Interestingly, expression of *Hd27-vank1* mRNA was weak in High Five cells, less abundant than Hd4 in the Ld-652 cell line and undetectable in the C6/36 cell lines. These findings suggest species-specific expression of *Hd27-vank1* mRNA that may possibly be related to the successful parasitism of *Spodoptera* hosts. By contrast, *Hd47-vank2* was detected at a fairly low level in all conditions tested. It is thus possible that expression of this gene is restricted to certain tissue types or hosts that were not tested in this study.

Expression patterns of HdIV *vankyrins* in both *Spodoptera* species differed noticeably between hemocytes and fat body. In *S. frugiperda*, almost all *vankyrins* were mainly expressed in hemocytes. Conversely, in *S. littoralis,* all HdIV *vankyrin* mRNAs, except for *Hd27-vank1*, tended to be more abundant in the fat body. The preferential expression in hemocytes or fat body has also been observed for some *Microplitis demolitor* bracovirus (MdBV) *vankyrin* genes [21] and CsIV *vankyrin* genes [12], but within a single host species. However, their functional roles in the different tissues remain unclear. The reasons for the differences in distribution of HdIV *vankyrin* mRNAs between *S. frugiperda* and *S. littoralis* are also unknown. Both species are successfully parasitized by *H. didymator* in laboratory conditions, causing severe physiological changes in both species, including developmental arrest, suppression of cellular and humoral immunity, and decreases in both host growth and hemocyte abundance [24]. However, only *S. littoralis* is the natural host of this parasitoid wasp and like *H. didymator* is naturally distributed in Europe. By contrast, *S. frugiperda*, which is found in North America, has not evolved in close association with HdIV. One possible explanation for the preferential expression in fat body of *S. littoralis* larvae may be specificity acquired during the co-evolution of HdIV and its natural host. Further identification of host factors involved in regulation of *vankyrin* expression and of host targets may help to elucidate this species-specific expression pattern.

The expression profiles of the *vankyrin* genes differed between HdIV-injected and parasitized *S. frugiperda* larvae, except for *Hd27-vank1* which was strongly expressed in both conditions. We used different larval stages for these two experiments: third-instar larvae were used for parasitism, and fifth-instar larvae were injected with HdIV. Therefore, it is possible that HdIV *vankyrin* members are regulated by a diverse set of host factors that are themselves differentially expressed during development of *S. frugiperda* larvae. Comparison of HdIV *vankyrin* profiles between HdIV-injected and parasitized third-instar larvae would help to answer this question, although the quantity of virus injected by the wasp remains unknown. Alternatively, *vankyrin* family genes may be regulated by maternal factors co-injected with the virus particles during oviposition, for example venom proteins or ovarian proteins. Regulation of this type has been already described for the *Cotesia rubecula* bracovirus (CrBV), the gene expression of which in host hemocytes is dependent on venom products [25]. Note also the amount of HdIV virus in injected calyx fluid and that naturally injected during parasitization are undoubtedly very different, which may explain some of the differences in *vankyrin* expression profiles observed.

To assess potential relationships between the *vankyrin* expression pattern and the host spectrum, we compared the expression profiles of HdIV *vankyrins* in a number of insect cell lines. First, the capacity of HdIV to infect these cells was evaluated by measuring the abundance of HdIV segments in the cytosol (after entry into the host cells) and in cell nuclei (where the viral DNA is transcribed). We found that only 8.3–12.4 % of the *vankyrin*-bearing segments in the calyx fluid could be recovered in lepidopteran cells indicating that a low proportion of virus particles crosses the plasma membrane. Even less HdIV crossed the membrane of mosquito cells, suggesting that this is a limiting step. The mechanisms governing the entry of PDV particles into the cytosol of the host cells are not well understood. The involvement of phagocytosis has been demonstrated only in hemocytes, whereas for other tissues a mechanism of membrane fusion involving the host cell plasma membrane and the particle inner membrane has been proposed [26]. Thus the low efficiency of virus entry into the cytosol of the mosquito cells may be because the particles bind poorly to the receptor(s) at the plasma membrane or because the mechanisms of membrane fusion are inefficient.

For each cell line, the relative proportions of the various DNA segments in the cytosol were generally consistent with the relative abundances of the segments in the calyx fluid. Conversely and surprisingly, infected lepidopteran cell lines showed marked differences in these relative ratios between the cytosol and nuclei. These observations suggest a mechanism of selection acting at the nuclear entry step or within the nucleus that may regulate the copy number of particular segments in the nucleus of host cells. At present little is known about genome packaging and nuclear entry of the PDVs. The current understanding of the packaging step is based principally on extrapolation from studies with BVs, for which it has been shown that intact nucleocapsids contain a single DNA segment [27], [28]. The situation for IV is less clear. Whether single or several DNA molecules are encapsidated in nucleocapsids is not known, although the size of IV virions is large enough to contain the complete set of DNA segments [27], [29]. Also, the strategy used by PDVs to gain access to the nucleus of host cells and deliver their genome into the nucleus remains elusive. Nucleocapsids of HfIV have been observed in the nuclei of host tissues [26], [30], despite the diameter of HfIV virions being above the upper particle diameter limit for transport through nuclear pore complexes by passive diffusion [31]. Electron microscopy has revealed the release of nucleocapsids of *Cotesia melanoscela* BV into the cytoplasm, followed by uncoating at nuclear pores and complete genome release into the nucleus of host cells [32]. It is not known whether these mechanisms are shared by all IVs and BV. Due to this lack of knowledge, it is difficult to explain the differences in relative abundance of the four HdIV segments between cytosol and nuclei in Noctuidae cells. There are various possible explanations, including the selective nuclear entry of DNA segments or HdIV nucleocapsids if, like HfIV, uncoating of HdIV takes place in the cytoplasm. Alternatively, there may be a selective degradation of nucleocapsids or released genomic segments either in the cytoplasm or nucleus. Further work is needed to answer these questions. Studies with MdBV have shown no variations of the relative abundance of genomic segments between calyx fluid and host tissues [28]. However, these experiments used total cellular extracts, and not isolated cell compartments. When we compared the respective abundance of the four HdIV segments between calyx fluid and total cellular extracts (sum of values for cytosol and nucleus) for each lepidopteran cell line, we did not observe any differences. Ours is thus the first report describing an unexpected level of control of the copy number of PDVs segments in the infected host, a phenomenon that may be important for the strength of expression of the corresponding genes.

We found evidence that host factors regulate the expression profiles of HdIV *vankyrin* genes in each insect cell line. It is difficult to appreciate the relevance of *in vitro* events to what happens *in vivo*, however it has been shown that polydnavirus transcription occurs *in vitro*, with a spectrum of transcripts similar to that *in vivo* [32], [33]. Thus, our findings for HdIV *vankyrin* expression in the various cell lines probably reflect the situation *in vivo*. A significant issue is the origin of the cell lines, as this may be important for determining whether the differences in the expression patterns observed are due to the species or the tissue of origin of the cells. Our *in vitro* transcriptional analysis of *vankyrin* genes is consistent with a pattern of species-specific expression potentially related to the success of parasitism in different hosts. As far as we are aware, the capacity of *H. didymator* to parasitize *L. dispar* and *T. ni* is not known. This parasitoid is a generalist wasp that parasitizes and develops in several noctuid species. *T. ni* is found in Europe in the same areas as *H. didymator* and, thus, is susceptible to be an alternative host for *H. didymator*. By contrast, the permissiveness of *L. dispar*, a Lymantriidae species also found in Europe, remains to be demonstrated. We show that *Hd27-vank1* is the most strongly expressed *vankyrin* gene in *Spodoptera* larvae and cells, suggesting that it may have specific and pleïotropic functions during parasitization of these species. More extensive studies are needed to elucidate the specific function of the *Hd27-vank1* protein during parasitism of *Spodoptera* larvae.

## Materials and Methods

### Insects and cell lines

*S. frugiperda* and *S. littoralis* were obtained from a laboratory colony maintained in stable conditions (25°C; 75±5 % relative humidity; 16 h light: 8 h dark photoperiod) and reared on a semi-synthetic diet. *H. didymator* parasitoids were reared on *S. frugiperda* at 27°C with a 16 h light: 8 h dark photoperiod. Five insect cell lines were used: Sf9 cells (ATCC CRL 1711) from *S. frugiperda*, Sl2b from *S. littoralis* hemocytes [34], High Five™ cells (BTI-TN-5B1-4) (Invitrogen) from *Trichoplusia ni* egg cell homogenates, Ld-652 (IPLB-Ld 652) from *Lymantria dispar* ovarian cells [35] and C6/36 cells (ATCC CRL-1660 FL) from *Aedes albopictus* whole larvae.

### Sequence analysis

For sequencing of the HdIV genome, dsDNA was extracted from purified viral particles as previously described [34]. The circular DNA molecules were then sequenced by the Genoscope as described in [16]. *Vankyrin* genes were identified by Blastx similarity searches against the NCBI nr database. Open reading frames were identified using KAIKOGAAS (*kaikogaas.dna.affrc.go.jp/usr/*). The molecular mass of proteins was estimated with the ExPASy proteomics server. Sequence Identity Matrices and multiple sequence alignments carried out using the CLUSTALW program were obtained from BioEdit (v7.0.5). The Simple Modular Architecture Research Toll (SMART) program (http://smart.embl-heidelberg.de/) was used to predict structural domains in amino acid sequences. The phylogenetic analysis was performed on the www.phylogeny.fr platform [36]. A multiple alignment was generated with MUSCLE (v3.7) and treated with Gblocks (v0.91b). The unrooted tree was reconstructed using the maximum likelihood computation implemented in the PhyML program (v3.0 aLRT) and drawn with TreeDyn [37].

### Virus preparation

Calyx fluid containing HdIV was prepared from *H. didymator* females as described [34]. Briefly, ovaries from about 20 female wasps were dissected in PBS, placed in a 1.5-ml microfuge tube and homogenized by several passages through a 23-gauge needle. The resulting suspension was passed through a 0.45-mm pore-size cellulose acetate filter and adjusted to 25 wasp equivalents per ml. One wasp equivalent (weq) is defined as the amount of HdIV collected from the ovaries of a single adult female. Filter-purified HdIV was stored at 4°C and used within 24 h.

### Parasitization, HdIV injection and cell infection

For parasitization, *H. didymator* female wasps and third-instar *S. frugiperda* larvae were placed in a dish at room temperature at a roughly 2∶1 (wasp:host) ratio. Only larvae that were parasitized once were retained for further analysis. Five larvae were sampled at 30 min, 1, 2, 6, 12 and 24 h post-parasitism. Total RNA was extracted and cDNA was synthesized as described below. For tissue-specific expression, total RNA from haemolymph, fat body, nerve cords and digestive tracts was isolated from a pool of 15 parasitized larvae. The haemolymph was collected from larvae punctured with syringe needle and transferred immediately into the RLT lysis buffer of the RNeasy Mini kit (Qiagen). Fat body, nerve cords and digestive tracts were then dissected out under a stereo light microscope and placed directly into RLT buffer for disruption and homogenization. To compare the expression profiles of HdIV *vankyrins* between *S. frugiperda* and *S. littoralis*, we injected 0.5 weq of HdIV into fifth-instar larvae (1-day-old). Larvae injected with PBS buffer served as negative controls. Five individuals were treated for each condition. Cells and tissues from HdIV- or PBS-injected fifth-instar *S. frugiperda* and *S. littoralis* larvae were collected 24 h post-injection as described for parasitized *S. frugiperda* larvae, except for hemocytes that were collected by centrifugation at 800 x *g*, washed in PBS and resuspended in RLT. Total RNA was extracted from the prepared tissue samples. For cell line infections, Sl2b, Sf9, High Five and C6-36 cells were seeded at 1.10^6^ cells/well and Ld-652 at 2.10^5^ cells/well and infected with 0.5 weq of HdIV. After 2 h infection, the inoculum was removed and the cell monolayers were washed two times with PBS. For qRT-PCR experiments, cells were cultured in their respective medium and total RNA was extracted at 24 h post-infection. For the quantification of HdIV DNA segments, two-hour HdIV-infected cells were immediately centrifuged at 1,500 x *g* at 4°C and cytoplasmic and nuclear DNA was extracted (see below).

### RNA isolation and cDNA preparation

Total RNA was isolated from various tissue samples and cell types with the RNeasy purification kit (Qiagen) according to the manufacturer's protocol. RNA samples were stored at −80°C until use. RNA samples were treated with the Turbo DNA-free Kit (Ambion) and the absence of contaminating DNA was verified by PCR. The total RNA concentration was estimated using the nanodrop ND-1000 spectrophotometer. Total RNA quality was estimated by 1% agarose gel electrophoresis or by Agilent 2100 bioanalyzer (Agilent Technologies, Santa Clara, California). Oligo(dT)-primed cDNA synthesis reactions were performed using Superscript III RNase H Reverse Transcriptase (Invitrogen) and the total RNA extracts.

### DNA extraction

HdIV DNA was extracted from calyx fluid with a QIamp DNA Mini Kit (Qiagen) and quantified using the nanodrop ND-1000 spectrophotometer. Sf9, Sl2b, Ld-652, and High Five™ cells were infected with the same calyx fluids, while C6/36 cells were infected with three other calyx fluids. For extraction of HdIV DNA from infected cells, cells were centrifuged at 800 x *g* at 4°C, re-suspended in extraction buffer A (20 mM HEPES pH 7.0, 0.15 mM EDTA, 0.15 mM EGTA, 10 mM KCl, 3 mM DTT, 1 % NP-40, 300 mM sucrose, 0.3 mM Spermidine), vortexed for 5 sec and centrifuged at 1,500 x *g* at 4°C for 5 min. Supernatants were transferred to a clean tube, centrifuged at 16,000 x *g* for 20 min, and the supernatants (the cytoplasmic extracts) were recovered and stored at −80°C. Pellets were re-suspended in extraction buffer B (10 mM HEPES pH 8.0, 0.1 mM NaCl, 25 % glycerol, 0.1 mM EDTA pH 8.0, 3 mM DTT and 0.3 mM Spermidine) and centrifuged at 2,000 x *g* at 4°C for 5 min. The resulting pellets were each re-suspended in extraction buffer C (10 mM HEPES pH 8.0, 0.4 mM NaCl, 25 % glycerol, 0.1 mM EDTA pH 8.0, 3 mM DTT and 0.3 mM Spermidine) and centrifuged at 10,000 x *g* at 4°C for 5 min. The resulting supernatants (the nuclear extracts) were then recovered and stored at −80°C. The cytoplasmic and nuclear DNA extracts were used for absolute quantification as described below.

### Quantitative real-time PCR

The PCR primers specific for each *vankyrin* gene and species-specific housekeeping genes used as internal controls were designed with Primer Express® software (v2.0.0). All primers used are listed in Table S1. As the sequences of *Hd29-vank1* and *Hd47-vank1* differ at only two nucleotide positions, a common primer pair was used to amplify both sequences; *Hd29/47-vank1* primers are assumed to not distinguish between the two genes. Each qPCR experiment consisted of triplicate technical runs for three independent RNA preparations per condition.

The mRNAs for HdIV *vankyrins* in the parasitized *S. frugiperda* whole larvae were assayed by quantitative real-time RT-PCR on an ABI Prism 7000 real-time PCR System (Applied Biosystems) using the Platinum® SYBR® Green qPCR SuperMix-UDG with ROX (Invitrogen). The amplification was carried out in a 25-µl PCR volume containing 20 ng of cDNA, 400 nM (each) forward and reverse primers, and 12.5 ìl PCR Master Mix. Each PCR amplification was performed under conditions of 95°C for 2 min, followed by 40 cycles of amplification (95°C for 15 s, 60°C for 1 min). Species-specific housekeeping genes [38] were used for normalization of the results.

Gene expression analyses of HdIV *vankyrins* in different tissues of parasitized larvae and HdIV-injected *Spodoptera* larvae were performed with the LightCycler Instrument (Roche Applied Science, Meylan, France) using the LightCycler® 480 SYBR Green I Master kit (High Resolution Melting Master). The amplifications were performed in a 384-well plate in a final volume of 10 µl containing 20 ng diluted cDNA sample, 5 µl Master Mix (2X), and 400 nM each primer. Amplification was performed as follows: 95°C for 10 min, followed by 45 cycles of 95°C for 5 s and 60°C for 30 s. Dissociation curve analysis was performed at the end of each PCR to ensure that only one product was amplified in each reaction. LightCycler software (LightCycler® 480 Software release 1.5.0 (1.5.0.39)) was used for data analysis. Results are reported as the amount of each *vankyrin* mRNA relative to that of housekeeping mRNAs, expressed as the mean ± standard deviation of the mean. A linear standard equation created from cDNA samples and mean crossing point (CP) values was obtained from standards. For the reliability of the qPCR, in run PCR efficiencies from each pair of primers were calculated and used for creation of relative standard curves. Starting quantities of cDNA samples were calculated from this equation, normalized to species-specific housekeeping genes and used to estimate the mean relative quantity of each sample.

Quantification of DNA segments and copy number of mRNA molecules in HdIV-infected cell lines were performed using absolute quantitative real-time PCR, with the LightCycler Instrument (Roche Applied Science, Meylan, France) using the LightCycler® 480 SYBR Green I Master kit (High Resolution Melting Master). The amplifications were performed in a 384-well plate in a final volume of 10 µl containing 6 ng diluted DNA or cDNA samples, 5 µl Master Mix (2X), and 400 nM each primer. Amplification was performed as follows: 95°C for 10 min, followed by 45 cycles of 95°C for 5 s and 60°C for 30 s. For HdIV segments quantification, oligonucleotide primer pairs (Table S1) were designed for specific amplification of portions of each HdIV segment containing gene. Segments Hd31, Hd4, hd27 and Hd47 were selected for this experiment. Standard 35-cycle PCRs (10 min of 95°C hot-start denaturation, 30 s of 95°C denaturation, 40 s of 58°C annealing, 1 min of 72°C elongation, 7-min 72°C final extension) were performed with DNA pools to amplify the viral genes using Go *Taq* flexi DNA Polymerase (Promega). Products were separated on 1 % agarose gels and visualized by ethidium bromide staining. The resulting cDNAs were purified with the MinElute PCR purification kit (Qiagen), quantified with the nanodrop ND-1000 spectrophotometer and used for the creation of absolute standard curves. For HdIV *vankyrin* quantification in the infected cell lines oligonucleotide primer pairs were designed for specific amplification of each gene. Standard PCRs were performed with DNA pools to amplify the viral genes. Products were separated, visualized and purified as described above and used for the creation of absolute standard curves.

### Statistical analysis

Student's *t*-test was used to determine the statistical significance of differences between parasitized and non-parasitized third-instar *S. frugiperda* larvae or HdIV-injected and PBS-injected fifth-instar larvae of *Spodoptera* spp. The Kruskal-Wallis test was used to determine the statistical significance of differences between the abundances of HdIV segments. P-values of less than 0.05 were considered to be statistically significant. All statistical analyses were performed using the R software (http://www.r-project.org/).

## Supporting Information

Table S1 **Oligonucleotides used for quantitative real-time PCR experiments.**
(DOC)
