# Supplementary material for: Multigenic Families in Ichnovirus: A Tissue and Host Specificity Study through Expression Analysis of Vankyrins from Hyposoter didymator Ichnovirus
Source: PLoS One. 2011 Nov 8;6(11):e27522. doi: 10.1371/journal.pone.0027522 (PMC3210807; doi:10.1371/journal.pone.0027522)
Supplement: Table S1 — Oligonucleotides used for quantitative real-time PCR experiments. (DOC) [file pone.0027522.s001.doc]

**Table S1.** Oligonucleotides used for quantitative real-time PCR experiments.

|  | **Gene** | **Species** | **Forward primer (5'-3')** | **Reverse primer (5'-3')** |
| --- | --- | --- | --- | --- |
| vankyrins | Hd4-vank1 | HdIV | ACCTGAATTGGCAACACGGA | CGGTTGTATGAAGCACGGTGT |
|  | Hd47-vank1 | HdIV | GTGATCGAGCTACTGCGGG | ATCGTCCCTTGCATCCAGAT |
|  | Hd47-vank2 | HdIV | CCATCCTGGCAGTGGAGAAC | CTGCGACGTGGATACAGGTG |
|  | Hd47-vank3 | HdIV | CTTGAGTGTTTGGCGTGGG | TGCTGACACAACCACTCGACA |
|  | Hd47-vank4 | HdIV | ATGAGTGTTTGGCGTGGTGA | CTGCTGACACAACCACTCTGC |
|  | Hd47-vank5 | HdIV | ATCTCTCAGGCGCAACCGT | TCCGTTTGTTCTGCTCCATG |
|  | Hd27-vank1 | HdIV | AGAGGAGTGAAGGCAATCCA | ATTGCACCAGTTCGTGATCG |
|  | Hd31-vank1 | HdIV | GCCGTACGAAATGGCATACA | AGGATGTCCATGATACGGTGG |
| House-keeping genes | COI | A. albopictus | GGAACGGTTTATCCTCCCCTT | CAACTGAAGCCCCAGCATG |
|  | Rpl32 | A. albopictus | CCGAAACGTATTGACAAGCGA | CAGGTACTGACCCTTGAAGCG |
|  | Elf1 | L. dispar | ATGCCTTGGTTCAAGGGATG | TTCAGCTTTGCCTTCCTTGC |
|  | β-actin | L. dispar | CTCCACCTCCCTCGAGAAGTC | GTGATGACCTGACCGTCGG |
|  | GADPH | S. littoralis | GTCATCATCTCTGCCCCCAG | GACACCAACCACGAACATGG |
|  | Elf1 | S. littoralis | CAGCTGCTGTCGCTTTCGTA | ACATGTTGTCTCCGTGCCAG |
|  | ATP Synthase | S. frugiperda | GCTGGTTCGTTCCTTGAGCA | GCCTTAACGCGTTCGCTTGC |
|  | Ubiquitin E2 | S. frugiperda | ACTTGTGGCCCGCATACACT | GGATCGGCACAATAAATGGG |
|  | Elf1 | T. ni | AGGAGGCTGTACCCGGAGAC | CGGAGACGTTCTTGACGTTGA |
|  | GADPH | T. ni | GTCATCATCTCTGCCCCCAGT | GACACCAACGACGAACATGG |
